# Open Application of Statistical and Machine Learning Models to Explore the Impact of Environmental Exposures on Health and Disease: An Asthma Use Case

**DOI:** 10.3390/ijerph182111398

**Published:** 2021-10-29

**Authors:** Bo Lan, Perry Haaland, Ashok Krishnamurthy, David B. Peden, Patrick L. Schmitt, Priya Sharma, Meghamala Sinha, Hao Xu, Karamarie Fecho

**Affiliations:** 1 UNC Highway Safety Research Center, University of North Carolina at Chapel Hill, Chapel Hill, NC 27599, USA; lan@hsrc.unc.edu; 2 Department of Statistics and Operations Research, University of North Carolina at Chapel Hill, Chapel Hill, NC 27599, USA; jigsandreels@gmail.com; 3 Renaissance Computing Institute, University of North Carolina at Chapel Hill, Chapel Hill, NC 27517, USA; ashok@renci.org (A.K.); plschmittt@gmail.com (P.L.S.); priyash@renci.org (P.S.); xuh123456@outlook.com (H.X.); 4 Department of Computer Science, University of North Carolina, Chapel Hill, NC 27599, USA; 5 Division of Allergy, Immunology and Rheumatology, Center for Environmental Medicine, Asthma & Lung Biology, University of North Carolina at Chapel Hill, Chapel Hill, NC 27599, USA; david_peden@med.unc.edu; 6 Department of Pediatrics, School of Medicine, University of North Carolina at Chapel Hill, Chapel Hill, NC 27599, USA; 7 Oregon State University, Corvallis, OR 97331, USA; sinham@oregonstate.edu; 8 Copperline Professional Solutions, LLC, Pittsboro, NC 27312, USA

**Keywords:** open data, open science, machine learning, conditional random forest, conditional tree, biostatistics, generalized linear model, asthma, epidemiology, public health

## Abstract

ICEES (Integrated Clinical and Environmental Exposures Service) provides a disease-agnostic, regulatory-compliant approach for openly exposing and analyzing clinical data that have been integrated at the patient level with environmental exposures data. ICEES is equipped with basic features to support exploratory analysis using statistical approaches, such as bivariate chi-square tests. We recently developed a method for using ICEES to generate multivariate tables for subsequent application of machine learning and statistical models. The objective of the present study was to use this approach to identify predictors of asthma exacerbations through the application of three multivariate methods: conditional random forest, conditional tree, and generalized linear model. Among seven potential predictor variables, we found five to be of significant importance using both conditional random forest and conditional tree: prednisone, race, airborne particulate exposure, obesity, and sex. The conditional tree method additionally identified several significant two-way and three-way interactions among the same variables. When we applied a generalized linear model, we identified four significant predictor variables, namely prednisone, race, airborne particulate exposure, and obesity. When ranked in order by effect size, the results were in agreement with the results from the conditional random forest and conditional tree methods as well as the published literature. Our results suggest that the open multivariate analytic capabilities provided by ICEES are valid in the context of an asthma use case and likely will have broad value in advancing open research in environmental and public health.

## 1. Introduction

Asthma and related pulmonary disorders are exquisitely sensitive to environmental exposures. For instance, numerous studies have found an association between exposure to airborne particulate matter and increased risk of asthma and asthma exacerbations [1,2]. Likewise, exposure to major roadways or highways, often used as a proxy for airborne pollutant exposure, also is associated with asthma exacerbations [3,4]. Demographic and clinical factors likewise influence the clinical course among patients with asthma. For instance, asthma exacerbations are more common among African Americans than Caucasians, females compared to males, and patients who are obese compared to those who are normal weight [5,6].

Studies on the impact of environmental exposures and other factors on asthma and related pulmonary disorders are commonly examined at the population level, using a variety of epidemiological approaches and models [7]. Less common are studies at the level of the individual, which support more robust methods and reduce confounding and other spurious limitations of population-based methods. The dearth of research on the health impacts of individual-level environmental exposures relates, in part, to the numerous regulatory, sociologic, and technical constraints surrounding the use of sensitive patient data. While these restrictions are essential to protect patient privacy and respect institutional concerns, they often pose challenges to research [8,9,10].

We developed the Integrated Clinical and Environmental Exposures Service (ICEES) to overcome these challenges by supporting open research on the health impact of environmental exposures at the level of the individual [11]. Specifically, ICEES provides a disease-agnostic, regulatory-compliant approach for openly exposing and analyzing clinical data (e.g., electronic health records, survey data) that have been integrated at the patient level with environmental exposures data. ICEES has been validated in the context of a driving use case on asthma, in which we demonstrated associations between asthma exacerbations and race, sex, obesity, and airborne pollutant exposure [11,12,13,14,15]. The service itself is disease-agnostic. Indeed, we have extended ICEES to support additional use cases on drug-induced liver injury, primary ciliary dyskinesia, and coronavirus infection.

ICEES was designed initially to support dynamic cohort creation and basic analytic functionalities, such as summary statistics and chi-square tests in an open yet regulatory-compliant manner. Herein, we describe the application of three machine learning or statistical methods to ICEES data using a new feature that supports the generation of multivariate tables using the basic functionalities available in ICEES [16]. We applied a conditional random forest (CRF) method, a conditional tree (CTree) method, and a generalized linear model (GLM) to data on an ICEES cohort of patients with asthma and related pulmonary conditions. We examined seven predictors of asthma exacerbations, as defined by emergency department (ED) or inpatient visits for respiratory issues: sex, race, prednisone, obesity, exposure to particulate matter ≤ 2.5 µm in diameter (PM_2.5_), and residential distance from a major roadway or highway. Finally, we compare model results and discuss key considerations when using ICEES for open multivariate analysis and applying different multivariate methods.

## 2. Materials and Methods

All study procedures were approved by the Institutional Review Board at the University of North Carolina at Chapel Hill (protocol #16-2978).

### 2.1. Overview of ICEES Open Multivariate Approach

Briefly, ICEES supports several basic analytic capabilities that allow users to explore the underlying data while maintaining regulatory compliance and abiding by institutional restrictions. Users can access the ICEES open application programming interface (OpenAPI) using either a Swagger interface or by way of programmatic command-line calls. Key features of ICEES are the ability to dynamically create cohorts and generate bivariate contingency tables with cell frequencies of patients and associated chi-square statistics and probability calculations returned to users. We recently developed an open approach that leverages these capabilities in such a way as to maintain contingencies between feature variables and generate a multivariate table with patient-level data derived from electronic health records and public sources of environmental exposures data [16]. Each row in the table represents data on an individual patient, and each column header represents a select feature variable. All data are binned or recoded so as to protect patient privacy. For instance, data on airborne pollutants are binned such that only abstracted values (e.g., 1, 2, …) representing ranges of exposure levels are returned to users. The open multivariate method introduces a certain amount of data loss due to the regulatory limitations of ICEES; for example, ICEES does not allow users to create cohorts of 10 or fewer patients. However, we verified that we can apply the open multivariate approach to generate a seven-feature multivariate table with an acceptable amount of data loss. We then applied a GLM to the data and generated results that replicated prior work by our group and others (e.g., [11]).

### 2.2. Data Source and Multivariate Table Generation

We focused on an ICEES cohort of *N* = 165,904 patients from UNC Health with asthma or related conditions (see [11] for details). Our use-case goal was to examine the impact of select demographic features and environmental exposures on asthma exacerbations. We examined seven independent variables: sex, race, prescriptions for prednisone, diagnosis of obesity, exposure to airborne particulates, residential proximity to a major roadway or highway, and residential density. These variables were selected on the basis of our prior work [11,14,15]. The annual number of ED or inpatient visits for respiratory issues was considered the primary outcome measure and indicator of asthma exacerbations. 

**Figure 1:**
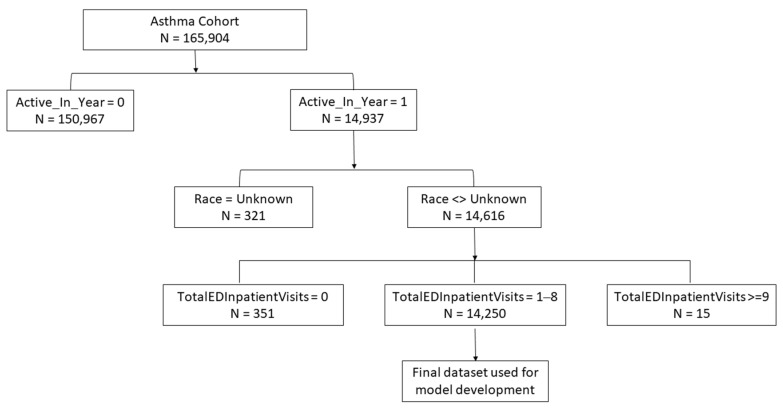
Flow chart depicting the sequential data processing steps taken to generate a final ICEES multivariate dataset for model development.

We performed a number of processing steps to clean the data and correct for imbalances (Figure 1). The imbalance in the annual number of ED or inpatient visits for respiratory issues was expected and due to the fact that few patients, including those with diagnosed respiratory disease, visit an ED or are admitted to a hospital due to respiratory issues in any given year. To adjust for the imbalance, we filtered the data to select only those patients who were “active” in calendar year 2010 (i.e., the first year of data available for the cohort), meaning that they had one or more visits to UNC Health during that year. The filtering step was intended to address the imbalance and enrich the dataset for those patients who were active within the healthcare system by excluding patients who were not active for any number of reasons (e.g., death, change in healthcare provider). Filtering by the variable Active_In_Year = 1 (yes) reduced the dataset to *N* = 14,937. We then excluded variable levels that skewed the distribution: patients whose race was unknown (*N* = 312) and patients without any ED or inpatient visits for respiratory issues (*N* = 351) or with a large number (>8) of ED or inpatient visits for respiratory issues (*N* = 15). These groups were excluded to further reduce imbalances in the data and overdispersion. For similar reasons, we collapsed racial categories with small numbers of patients (i.e., Asian, Native Hawaiian/Pacific Islander, American/Alaskan Native, Other), and we collapsed contiguous bins for MaxDailyPM2.5Exposure_StudyMax from five to two. 

The final dataset was comprised of *N* = 14,250 patients. Summary statistics are provided in Table 1. The majority of patients were female (56.3%) and Caucasian (59.3%). Most patients (62.8%) lived ≥ 250 meters from a major roadway or highway, and 72.4% resided in a geographical location classified as rural. No patients were categorized as residing in an urbanized area of ≥ 50,000 patients. In terms of medications and diagnoses, 9.8% of patients had been diagnosed as obese, and 8.9% received at least one prescription for prednisone over the one-year study period. The majority of patients (67.6%) had one visit to the ED or an inpatient clinic for respiratory issues over the one-year period (range: 1–8; mean = 1.56 ± 1.03 [standard deviation]).

### 2.3. Analytic Approach

This study aimed to identify environmental exposures and other factors that predict the annual number of ED or inpatient visits for respiratory issues. The dependent variable was therefore the number of annual ED or inpatient visits, which is a discrete number or count. The independent variables were categorical: sex, race, prednisone use, diagnosis of obesity, exposure to airborne particulates, residential proximity to a major roadway or highway, and residential density. We applied three machine learning or statistical approaches to the generated ICEES multivariate feature table: CRF, CTree, and GLM. CRF [17,18,19,20] was first used to identify the most important predictor variables among the seven independent variables that were selected for analysis. CTree was then used to confirm the important predictor variables and explore possible interactions among them. Next, GLM, informed by the output from the CTree analysis, was used to model counts of annual ED or inpatient visits for respiratory issues in a regression context using Negative Binomial (NB) and Poisson regressions [21]. 

#### 2.3.1. CRF Analysis

Two different statistical packages were initially considered for analysis: randomForest and cforest within Package party in R [17,18,19,20,22,23,24]. The two packages are similar in that they use random subsets of the input data for recursive partitioning in order to develop multiple classification or regression trees. The aggregated result of the multiple trees is then used to estimate the importance of each predictor variable in modeling the dependent variable. There are, however, key differences between the two packages in terms of how they determine predictor variable importance. First, randomForest does not accurately account for correlation among predictor variables. The result is an overestimation of the importance of highly correlated predictors. The cforest algorithm attempts to overcome these shortcomings by invoking a conditional permutation-importance measure. Second, randomForest requires that continuous dependent variables are normally distributed. The dependent variable in the current study was a discrete (not continuous) variable, and it did not have a normal distribution. Given these considerations, we chose to use the cforest algorithm within Package party in R for the CRF analysis in order to determine the most important predictor variables. 

We selected cforest options to generate 1000 trees and apply a splitting criterion with α = 0.05. We also had to select between bootstrap sampling with replacement and subsampling without replacement. The former is the traditional method used in random forest analysis, but it has been shown to artificially introduce associations between predictor variables and introduce bias towards the selection of predictor variables with more categories [20]. Thus, we opted to select subsampling without replacement, and we adopted the suggested default setting in function cforest_unbiased(), where 63.2% of the data were randomly sampled without replacement and used as a training dataset, while the remaining 36.8% were used as a test dataset. 

#### 2.3.2. CTree Analysis

Having adopted the cforest algorithm for the CRF analysis, we chose to use the CTree algorithm within Package party in R for the CTree analysis in order to determine the most important interactions among predictor variables [17,18,19,20]. CTree analysis is a robust data-mining and data-analysis tool that automatically searches for important patterns and relationships and quickly uncovers hidden structures even within highly complex data. The method does not require any predefined underlying relationship between target (dependent variable) and predictors (independent variables), and it has been shown to be a powerful tool to find the more important predictor variables in relation to the target variable. CTree also provides the ability to determine the level and type of interactions between predictor variables without having to predefine such interactions. In addition, CTree can identify and explain complex patterns associated with asthma risk, and it can effectively handle multi-collinearity problems and observations with missing values. 

For the CTree analysis, a permutation test was used to evaluate the association between the predictor variables and the target or dependent variable, measured by a *p* value. We set the significance level at α = 0.05.

#### 2.3.3. GLM Analysis

The third approach that we applied was to fit a robust GLM, taking into account the results from the CTree analysis and exploring a few scenarios with or without interactions among predictor variables. Currently, the favored GLM models are NB and Poisson regressions. NB regression can be used to adjust for overdispersion with count data, whereas the Poisson regression cannot. Both modeling methods were explored and compared in this study. The final model was selected based on the model comparison criteria, which are the Akaike information criterion (AIC) [25,26] and the Bayesian information criterion, sometimes referred to as the Schwarz information criterion (BIC) [26,27,28]. The criteria were expressed as shown in Equations (1) and (2).

(1) AIC=2k−2 ln(L)

(2) BIC=k ln(n)−2 ln(L)

where:

*k* = number of free parameters in the model;

*n* = sample size; and

*L* = maximized value of the likelihood function.

For both comparison criteria, lower values indicate a better model fit. A larger maximized value of the likelihood function (*L*) produces a smaller value for both metrics. Likewise, fewer parameters in the model (*k*) produce a smaller value for both criteria. The difference in the two criteria is the magnitude of the penalty assigned for the number of parameters in the model. The AIC uses a multiplier of 2.0 and does not account for sample size. The BIC accounts for the size of the sample with the multiplier *ln*(*n*). The goal is to select the model with the best fit and the fewest parameters. Thus, the BIC was chosen as the preferred criterion for comparison of models; however, the AIC was considered when there was no or little change in the BIC. The magnitude of the change in these comparison criteria also was considered when choosing the best model fit, although this can be neglected if the change in both the AIC and the BIC is less than 2.0. The difference can be deemed as substantial if the change is between 2.0 and 6.0, and if the change is greater than 6.0, then the model with the lower values is strongly favored [26,27].

## 3. Results

### 3.1. CRF Analysis

**Figure 2:**
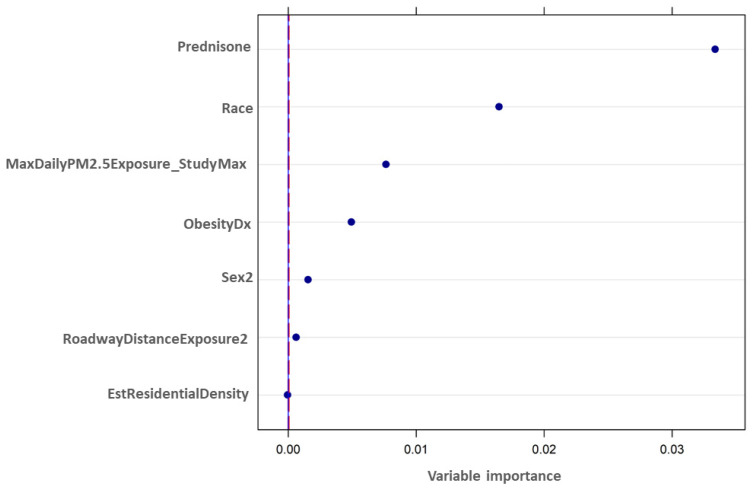
Predictor variable importance, as determined by CRF analysis. The cforest algorithm within Package party in R was applied to the ICEES multivariate dataset (see Figure 1 and Table 1) to determine the most important predictor variables in relation to the dependent variable of total annual ED or inpatient visits for respiratory issues. The vertical dashed line represents the significance threshold; values to the right of the line are significant. Variables are defined in Table 1.

The output from the CRF analysis using cforest, with annual total ED or inpatient visits for respiratory issues as the dependent or target variable, is shown in Figure 2. Variables were considered informative and important if their variable importance value was above the absolute value of the lowest negative-scoring variable. The rationale for this rule-of-thumb was that the importance of irrelevant variables varies randomly around zero [20]. The strongest predictor variable is shown at the top of the figure, with the other important predictor variables listed in decreasing order of importance. Six of the seven independent variables that were considered were identified as important predictors: Prednisone, Race, MaxDailyPM2.5Exposure_StudyMax, ObesityDx, Sex2, and RoadwayDistanceExposure2. EstResidentialDensity was not identified as an important predictor variable. Prednisone was the most important predictor variable, followed by Race and then MaxDailyPM2.5Exposure_StudyMax.

### 3.2. CTree Analysis

**Figure 3:**
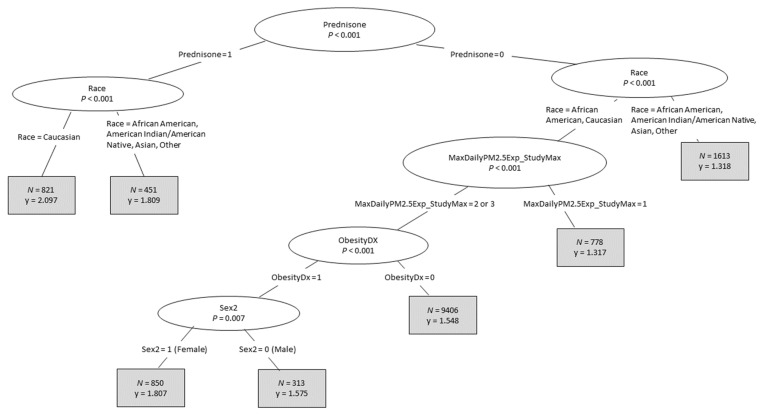
Predictor variable importance and interactions as determined by CTree analysis. The CTree algorithm within Package party in R was applied to the ICEES multivariate dataset (see Figure 1 and Table 1) to confirm the most important predictor variables determined by the CRF analysis in terms of relation to the dependent variable of total annual ED or inpatient visits for respiratory issues and to identify significant interactions between predictor variables. The enumeration levels for each variable are indicated on each branch of the tree. Variables are defined in Table 1.

With the important predictor variables identified through the CRF analysis, the CTree algorithm within Package party in R was used to develop conditional inference trees in order to confirm the important predictor variables and determine interactions between them. The CTree output lists the most important predictor variables at the top of the tree, with variable importance decreasing from top to bottom (Figure 3). The CTree analysis found that the top five most important predictors variables were the same as those from the CRF analysis and ranked in the same order from most important to least: Prednisone, Race; MaxDailyPM2.5Exposure_StudyMax, ObesityDx, and Sex2. The CTree analysis did not identify RoadwayDistanceExposure2 or EstResidentialDensity as significant predictor variables. 

In terms of interactions, CTree identified the following two-way interactions as significant: Prednisone*Race; Race*MaxDailyPM2.5Exposure_StudyMax; MaxDailyPM2.5Exposures_StudyMax*ObesityDx; and ObesityDx*Sex2. Significant three-way interactions were Predisone*Race*MaxDailyPM2.5Exposure_StudyMax; Race*MaxDailyPM2.5Exposure_StudyMax*ObesityDx; and MaxDailyPM2.5Exposure_StudyMax*ObesityDx*Sex2. Higher-order interactions were not considered due to poor model fit.

Closer inspection of the interactions revealed additional insights. For instance, in Figure 3, the mean number of annual ED or inpatient visits for respiratory issues when Prednisone = 1 was 2.097 for Caucasians and 1.809 for the combined “African American” and “Asian, Native Hawaiian/Pacific Islander, American/Alaskan Native, Other” group, indicating a greater impact of prednisone on the target or dependent variable among Caucasians than the other racial groups. When Prednisone = 0, the mean number of annual ED or inpatient visits for respiratory issues was significantly lower among the “Asian, Native Hawaiian/Pacific Islander, American/Alaskan Native, Other” group (1.318) than among the combined Caucasian and African American group (1.552). Among Caucasians and African Americans, with Prednisone = 0, the mean number of annual ED or inpatient visits for respiratory issues was higher among those with MaxDailyPM2.5Exposure_ StudyMax = 2 than those with MaxDailyPM2.5Exposure_StudyMax = 1. ObesityDx = 1 and Sex2 = 1 (female) imparted further risk or impact on annual ED or inpatient visits for respiratory issues. 

### 3.3. GLM Analysis

For development of the GLM model, the interactions identified from the CTree analysis output were considered in the context of four scenarios: 

Scenario 1 included all seven potential predictor variables;

Scenario 2 included all seven potential predictor variables plus the top two-way interaction from the CTree output, namely Prednisone*Race;

Scenario 3 included all seven potential predictor variables plus all significant two-way interactions from the CTree output, namely Prednisone*Race, Race*MaxDailyPM2.5Exposure_StudyMax, MaxDailyPM2.5Exposure_StudyMax*ObesityDx, and ObesityDx*Sex2; and

Scenario 4 considered all seven potential predictor variables plus the two-way interactions included under Scenario 3 plus all significant three-way interactions from the CTree output, namely Prednisone*Race*MaxDailyPM2.5Exposure_StudyMax, Race*MaxDailyPM2.5Exposure_StudyMax*ObesityDx, and MaxDailyPM2.5Exposure_StudyMax*ObesityDx*Sex2.

The above four scenarios were first explored using a NB regression. We found that the overdispersion parameter was 0 or close to 0, meaning that the number of annual ED or inpatient visits followed a simple Poisson distribution. Thus, Poisson regression was used instead of NB regression in the final GLM. Of note, a Poisson distribution also best adjusted for the imbalance in the data. 

The AIC and BIC metrics for the four scenarios were then compared, using both Poisson regression and Lasso Poisson regression, as shown in Table 2. The Lasso Poisson regression metrics were generally weaker than those for the Poisson regression. As such, we adopted Poisson regression for further model comparison. Scenario 2 had the lowest BIC and a relatively low AIC under Poisson regression. Thus, the GLM from Scenario 2 under Poisson regression was selected as the best fit model based on the evaluation criteria we applied.

The GLM results identified four significant predictor variables, ranked in order from greatest to lowest importance in terms of their estimated effect on the dependent variable: Prednisone, Race, MaxDailyPM2.5Exposure_StudyMax, and ObesityDx (Table 3). These results were largely consistent with the CRF and CTree analysis results, except that the GLM analysis did not identify Sex2 as a significant predictor variable, which the CRF and CTree analyses did. Interactions between prednisone and race also were as expected except for the estimated interaction between the “Asian, Native Hawaiian/Pacific Islander, American/Alaskan Native, Other” group and prednisone group, in which patients with Prednisone = 1 were predicted to have fewer annual ED or inpatient visits for respiratory issues than patients with Prednisone = 0, which was counter to expectations. Upon further examination of the data (Table 4), we found that the sample size for Race = Asian, Native Hawaiian/Pacific Islander, American/Alaskan Native, Other and Prednisone = 1 was small (*N* = 51), with all patients having had only one ED or inpatient visit for respiratory issues over the one-year study period. In contrast, for the other two racial groups, the mean annual number of ED or inpatient visits for respiratory issues with Prednisone = 1 was higher than the mean number with Prednisone = 0. 

As expected, the predicted standard deviation for the mean number of annual ED or inpatient visits was lower than the observed standard deviation. This likely reflects the fact that the predicted standard deviation estimates are smoothed values derived from the model. In addition, the model includes a relatively small number of predictor variables, all of which are categorical. Nonetheless, the GLM results were largely as expected and consistent with those of the CRF and CTree analyses.

## 4. Discussion

### 4.1. Summary of Results and Relation to Published Literature

We successfully used the ICEES OpenAPI to generate an eight-feature multivariate table by exploiting bivariate contingencies derived from the service’s dynamic cohort definition capabilities, bivariate functionalities, and programmatic API calls. We then applied three statistical methods—CRF, CTree, GLM—to evaluate the impact of select independent variables on the dependent variable of annual ED or inpatient visits for respiratory issues among patients with asthma and related conditions. Among seven potential predictor variables (sex, race, prescriptions for prednisone, diagnosis of obesity, exposure to airborne particulates, residential proximity to a major roadway or highway, and residential density) that were selected a priori on the basis of our prior results and those of other groups [1,2,3,4,5,6,11,12,13,15], we found five to be of significant importance using both the CRF and CTree analytic methods, namely prednisone, race, exposure to airborne particulates, obesity, and sex. Moreover, both machine learning methods ranked the predictor variables in the same order, with prednisone use as the most important predictor variable. CTree additionally identified several significant two-way and three-way interactions among the same variables: prednisone x race; race x airborne particulate exposure; airborne particulate exposure x obesity; obesity x sex; prednisone x race x airborne particulate exposure; race x airborne particulate exposures x obesity; and airborne particulate exposure x obesity x sex. When we applied a GLM model using all seven potential predictor variables and the top two-way interaction identified by the CTree analysis (prednisone x race), we identified four significant predictor variables, namely prednisone, race, airborne particulate exposure, and obesity, and all three methods ranked the variables in the same order, in terms of effect size. 

These results were largely as expected given previous findings by our group and others. However, there were exceptions. For instance, the final GLM did not identify sex as a significant predictor, which our group [12] and others [5,6] have found to be significant, with asthma exacerbations more common among females than males. However, the CRF and CTree methods did identify sex as a significant predictor, with asthma exacerbations more common among females than males. Similarly, the CTree and GLM methods did not identify close proximity to a major roadway or highway as a significant predictor of asthma exacerbations in contrast to the CRF method and findings by other groups [3,4]. We suspect that these differences relate to the fact that the asthma cohort was largely derived from rural areas. Indeed, we did not have any patients classified as residing in an urbanized area. Another unexpected finding was that asthma exacerbations were more common among Caucasians than among African Americans, which contradicts our prior findings [15] and those of other groups [5]. We suspect that this discrepancy relates to the fact that our institution’s racial category of “Caucasian” does not distinguish racial or ethnic subcategories, such as “Hispanic” or “Latino”. We are exploring approaches to distinguish racial and ethnic subcategories. One final unexpected finding was the GLM prediction that prednisone use decreases the likelihood of asthma exacerbations among American Indians/Alaskan Natives/Asians/Others. Prednisone is typically prescribed as an acute treatment for asthma exacerbations among patients with severe asthma; it is not typically used as a preventative treatment [29]. In the current study, we hypothesized a priori that prednisone use would be associated with an increase in the number of annual ED or inpatient visits for respiratory issues, which we found to be true in all racial categories except for the combined American Indian/Alaskan Native/Asian/Other group. We believe that the anomaly is due to a small sample size, but there may be other factors that we did not account for in the present study or that uniquely impact certain racial groups and/or treatment choices.

### 4.2. Service Considerations

ICEES has several features worth highlighting. First, the service is freely available and designed to democratize and accelerate open research on environmental determinants of health and disease. In contrast to epidemiological studies that focus on population-level correlations between environmental exposures and health-related metrics, such as healthcare utilization, ICEES supports individual-level research on demographic and environmental risk factors for health outcomes such as asthma exacerbations. As such, ICEES can be applied to identify individual predictors of morbidity and mortality, thus extending the utility of the service beyond research to potentially augment clinical decision making, guide public health policies, and contribute to other real-world applications. While the results presented herein focus on a cohort of patients with asthma and related respiratory conditions, ICEES itself is generalizable. Indeed, we are developing new ICEES instances to support open research on demographic and environmental determinants of risk and clinical course in drug-induced liver injury, primary ciliary dyskinesia, coronavirus infection, and other diseases and conditions. 

### 4.3. Model Considerations

The CRF, CTree, and GLM analyses mostly yielded consistent results, although we did find slight differences, primarily in terms of the number of predictors that were identified and their relative importance in relation to our dependent variable of asthma exacerbations. One advantage of machine learning methods, including random forest and conditional tree methods, is that distributional assumptions are not required. In contrast, the GLM method requires specification of the model and underlying distribution of the dependent variable. Given these methodological differences, the consistency across methods suggests that the final four predictors that we identified—prednisone, race, airborne particulate exposure, and obesity—are especially important in predicting asthma exacerbations. 

Other model considerations include the magnitude and directionality of outputs. While the CRF analysis identified important predictors of annual ED or inpatient visits for respiratory issues, the CRF method does not provide information on the magnitude or directionality of the effect. In contrast, the CTree method and the GLM model do provide information on the magnitude and directionality of the effect. Thus, results from the CTree method and the GLM model can be compared directly, and the results were largely consistent. Prednisone was found to produce the greatest effect on the dependent variable, followed by race, airborne particulate exposure, and obesity. In terms of directionality, prednisone use, Caucasian race, exposure to relatively high levels of airborne particulates, and a diagnosis of obesity predicted an increase in the annual number of ED or inpatient visits for respiratory issues.

Finally, the modeling work presented herein has limitations that should be considered when interpreting the results. Specifically, we focused on one year of data only and conducted the work as a feasibility study. As such, we did not perform a rigorous out-of-sample validation test or stringent assessment of model performance and accuracy. We did, however, identify consistent results across three distinct approaches, thus demonstrating feasibility. We now plan to conduct a more rigorous study using a larger dataset and multiple years of data. We expect this work to not only strengthen the rigor of our analyses but also allow us to move beyond replication of prior work to generate new insights and hypotheses.

## 5. Conclusions

We successfully used the open-source ICEES API to generate an eight-feature multivariate table and apply three analytic methods—CRF, CTree, and GLM—to identify the impact of select predictor variables on annual ED or inpatient visits for respiratory issues among patients with asthma and related conditions. Our results not only provided insights into demographic factors and environmental exposures that influence asthma exacerbations, but they suggest that the novel, open multivariate analytic capabilities provided by the disease-agnostic ICEES likely will have value in advancing and accelerating open research across many areas of environmental and public health.

## Figures and Tables

**Table 1:** Summary statistics for final ICEES multivariate dataset used for model development.

Feature Variable (ICEES Feature Variable Name)	Variable Enumeration	*N* (%)
Total Annual ED or Inpatient Visits for Respiratory Issues	1	9633 (67.6%)
	2	2677 (18.8%)
	3	1067 (7.5%)
	4	492 (3.5%)
	5	210 (1.5%)
	6	115 (0.8%)
	7	43 (0.3%)
	8	13 (0.1%)
Sex (Sex2)	0 (Male)	6231 (43.7%)
	1 (Female)	8019 (56.3%)
Race (Race)	Caucasian	8457 (59.3%)
	African American	4111 (28.9%)
	Asian, Native Hawaiian/Pacific Islander, American/Alaskan Native, Other	1682 (11.8%)
Prescription or Administration of Prednisone (Prednisone) ^1^	0 (No)	12,978 (91.1%)
	1 (Yes)	1272 (8.9%)
Diagnosis of Obesity (ObesityDx) ^2^	0 (No)	12,859 (90.2%)
	1 (Yes)	1391 (9.8%)
Maximum Daily PM_2.5_ Exposure, µg/m^3^ (MaxDailyPM2.5Exposure_StudyMax)	1 (6.58, 25.09)	878 (6.2%)
	2 (25.09, 98.76)	13,372 (93.8%)
Residential Distance to a Major Roadway or Highway, Meters (RoadwayDistanceExposure2)	1 (0–49)	1629 (11.4%)
	2 (50–99)	1008 (7.1%)
	3 (100–149)	878 (6.2%)
	4 (150–199)	922 (6.5%)
	5 (200–249)	861 (6.0%)
	6 (≥250)	8952 (62.8%)
Estimated Residential Density, Persons (EstResidentialDensity) ^3^	1 (0, 2500)	10,321 (72.4%)
	2 (2500, 50,000)	3929 (27.6%)
	3 (50,000, infinity)	0 (0.0%)
**TOTAL**	14,250	

Abbreviations: ED, emergency department; PM_2.5_, particulate matter ≤ 2.5 µm in diameter. ^1^ One or more prescriptions for prednisone over one-year study period. ^2^ One or more diagnoses for obesity over one-year study period. ^3^ US Census Bureau American Community Survey 2007–2011 estimated total population (block group), binned according to U.S. Census Bureau definitions (1, rural (0, 2500); 2, urban cluster (2500, 50,000); 3, urbanized area (50,000, inf).

**Table 2:** GLM scenario comparison.

Scenario ^1^	Poisson Regression		Lasso Poisson Regression	
	AIC	BIC	AIC	BIC
Scenario 1: main effects, no interaction terms	39,548	39,593	39,559	39,612
Scenario 2: main effects, top significant two-way interaction identified by CTree analysis	**39,531**	**39,592**	39,559	39,627
Scenario 3: main effects, all significant two-way interactions identified by CTree analysis	39,525	39,601	39,563	39,608
Scenario 4: main effects, all significant two- and three-way interactions identified by CTree analysis	39,520	39,618	39,544	39,604

Abbreviations: AIC, Akaike information criterion; BIC, Bayesian information criterion; CTree, conditional tree; GLM, generalized linear model. ^1^ Refer to text for complete description of each scenario. The final GLM model that we applied is described in Equation (3): Y = Exp(0.0916 + β_1_Race + β_2_MaxDailyPM2.5Exposure_StudyMax_cat + β_3_(Prednisone * Race) + β_4_ObesityDx) (3), where Y = predicted annual number of ED or inpatient visits for respiratory issues; β_1_ = estimated model parameters for Race; β_2_ = estimated model parameters for MaximumDailyPM2.5Exposure_StudyMax; β_3_ = estimated model parameters for interaction between Prednisone and Race; and β_4_ = estimated model parameters for ObesityDx. Bold font was used to highlight the metrics that were used to select the final model.

**Table 3:** Final GLM model output.

Parameter		Estimate	Standard Error	*p*-Value
Intercept		0.0916	0.0366	0.0122
Race = African American		0.1491	0.0254	<0.0001
Race = Caucasian		0.1685	0.0235	<0.0001
Race = Asian, Native Hawaiian/Pacific Islander, American/Alaskan Native, Other		0	0	.
MaxDailyPM2.5Exposure_StudyMax = 2		0.1869	0.0304	<0.0001
MaxDailyPM2.5Exposure_StudyMax = 1		0	0	.
ObesityDx = 1		0.0966	0.0217	<0.0001
ObesityDx = 0		0	0	.
Race = African American	Prednisone = 1	0.2062	0.0385	<0.0001
Race = African American	Prednisone = 0	0	0	.
Race = Caucasian	Prednisone = 1	0.294	0.0258	<0.0001
Race = Caucasian	Prednisone = 0	0	0	.
Race = Asian, Native Hawaiian/Pacific Islander, American/Alaskan Native, Other	Prednisone = 1	−0.2785	0.1417	0.0493
Race = Asian, Native Hawaiian/Pacific Islander, American/Alaskan Native, Other	Prednisone = 0	0	0	.

Abbreviation: GLM, generalized linear model.

**Table 4:** Observed versus predicted mean annual ED or inpatient visits for respiratory issues.

Parameter		*N*	Mean ^1^	Std Dev (Observed)	Std Dev (Predicted)
Race = African American		4111	1.58	1	0.14
Race = Caucasian		8457	1.61	1.11	0.18
Race = Asian, Native Hawaiian/Pacific Islander, American/Alaskan Native, Other		1682	1.31	0.64	0.07
MaxDailyPM2.5Exposure_StudyMax = 2		13372	1.58	1.06	0.18
MaxDailyPM2.5Exposure_StudyMax = 1		878	1.31	0.58	0.11
ObesityDx = 1		1391	1.73	1.05	0.17
ObesityDx = 0		12859	1.55	1.03	0.18
Race = African American	Prednisone = 1	400	1.91	1.11	0.07
Race = African American	Prednisone = 0	3711	1.54	0.98	0.08
Race = Caucasian	Prednisone = 1	821	2.1	1.42	0.1
Race = Caucasian	Prednisone = 0	7636	1.56	1.05	0.09
Race = Asian, Native Hawaiian/Pacific Islander, American/Alaskan Native, Other	Prednisone = 1	51	1	0	0
Race = Asian, Native Hawaiian/Pacific Islander, American/Alaskan Native, Other	Prednisone = 0	1631	1.32	0.64	0.05

Abbreviation: ED, emergency department; Std Dev, standard deviation. ^1^ Observed versus predicted mean annual ED or inpatient visits for respiratory issues are identical for the applied model, but standard deviation estimates differ.

## Data Availability

ICEES is freely available and can be accessed at: https://icees.renci.org:16340/apidocs#/.

## References

[1] Mirabelli M., Vaidyanathan A., Flanders W.D., Qin X., Garbe P. (2016). Outdoor PM2.5, Ambient Air Temperature, and Asthma Symptoms in the Past 14 Days among Adults with Active Asthma. Environ. Health. Perspect..

[2] Requia W.J., Adams M.D., Koutrakis P. (2017). Association of PM2.5 with diabetes, asthma, and high blood pressure incidence in Canada: A spatiotemporal analysis of the impacts of the energy generation and fuel sales. Sci. Total Environ..

[3] Pérez L., Lurmann F., Wilson J., Pastor M., Brandt S.J., Künzli N., McConnell R. (2012). Near-Roadway Pollution and Childhood Asthma: Implications for Developing “Win–Win” Compact Urban Development and Clean Vehicle Strategies. Environ. Health Perspect..

[4] Schurman S.H., Bravo M., Innes C.L., Jackson W.B., McGrath J.A., Miranda M.L., Garantziotis S. (2018). Toll-like Receptor 4 Pathway Polymorphisms Interact with Pollution to Influence Asthma Diagnosis and Severity. Sci. Rep..

[5] Keet C.A., McCormack M.C., Pollack C.E., Peng R.D., McGowan E., Matsui E.C. (2015). Neighborhood poverty, urban residence, race/ethnicity, and asthma: Rethinking the inner-city asthma epidemic. J. Allergy Clin. Immunol..

[6] Greenblatt R.E., Zhao E.J., Henrickson S.E., Apter A.J., Hubbard R.A., Himes B.E. (2019). Factors associated with exacerbations among adults with asthma according to electronic health record data. Asthma Res. Pract..

[7] Bind M.-A. (2019). Causal Modeling in Environmental Health. Annu. Rev. Public Health.

[8] Parija S.C., Mandal J., Acharya S. (2011). Ethics in human research. Trop. Parasitol..

[9] Wacker J., Kolbe M. (2016). The challenge of learning from perioperative patient harm. Trends Anaesth. Crit. Care.

[10] Lubarski B. Re-Identification of “Anonymized” Data. Georgetown Law Technology Review. https://www.georgetownlawtechreview.org/re-identification-of-anonymized-data/GLTR-04-2017.

[11] Fecho K., Pfaff E., Xu H., Champion J., Cox S., Stillwell L., Peden D.B., Bizon C., Krishnamurthy A., Tropsha A. (2019). A novel approach for exposing and sharing clinical data: The Translator Integrated Clinical and Environmental Exposures Service. J. Am. Med. Inform. Assoc..

[12] Ahalt S.C., Chute C.G., Fecho K., Glusman G., Hadlock J., Taylor C., Pfaff E., Robinson P.N., Solbrig H., Ta C. (2019). Clinical Data: Sources and Types, Regulatory Constraints, Applications. Clin. Transl. Sci..

[13] Fecho K., Ahalt S.C., Arunachalam S., Champion J., Chute C.G., Davis S., Gersing K., Glusman G., Hadlock J., Lee J. (2019). Sex, obesity, diabetes, and exposure to particulate matter among patients with severe asthma: Scientific insights from a comparative analysis of open clinical data sources during a five-day hackathon. J. Biomed. Inform..

[14] Pfaff E.R., Champion J., Bradford R.L., Clark M., Xu H., Fecho K., Krishnamurthy A., Cox S., Chute C.G., Taylor C.O. (2019). Fast Healthcare Interoperability Resources (FHIR) as a Meta Model to Integrate Common Data Models: Development of a Tool and Quantitative Validation Study. JMIR Med. Inform..

[15] Xu H., Cox S., Stillwell L., Pfaff E., Champion J., Ahalt S.C., Fecho K. (2020). FHIR PIT: An open software application for spatiotemporal integration of clinical data and environmental exposures data. BMC Med. Inform. Decis. Mak..

[16] Fecho K., Haaland P., Krishnamurthy A., Lan B., Ramsey S., Schmitt P.L., Sharma P., Sinha M., Xu H. (2021). Development and application of an open approach for multivariate analysis of integrated clinical and environmental exposures data. IMU.

[17] Hothorn T. Party: A Laboratory for Recursive Partytioning. http://cran.r-project.org/web/packages/party/index.html.

[18] Strobl C., Boulesteix A.-L., Zeileis A., Hothorn T. (2007). Bias in random forest variable importance measures: Illustrations, sources and a solution. BMC Bioinform..

[19] Strobl C., Boulesteix A.-L., Kneib T., Augustin T., Zeileis A. (2008). Conditional variable importance for random forests. BMC Bioinform..

[20] Strobl C., Malley J., Tutz G. (2009). An introduction to recursive partitioning: Rationale, application, and characteristics of classification and regression trees, bagging, and random forests. Psychol. Methods.

[21] Townes F.W. Review of Probability Distributions for Modeling Count Data. https://arxiv.org/abs/2001.04343v1.

[22] Brieman L., Cutler A. (2013). Random Forests. http://www.stat.berkeley.edu/~breiman/RandomForests/.

[23] Breiman L., Friedman J.H., Olshen R.A., Stone C.J. (2017). Classification and Regression Trees. Classification and Regression Trees.

[24] Liaw A., Wiener M. (2002). Classification and regression by randomForest. R News.

[25] Akaike H., Parzen E., Tanabe K., Kitagawa G. (1998). Information Theory and an Extension of the Maximum Likelihood Principle. Selected Papers of Hirotugu Akaike.

[26] Burnham K.P., Anderson D.R. (2002). Model Selection and Multimodel Inference: A Practical Information-Theoretic Approach.

[27] Raftery A.E. (1999). Bayes Factors and BIC. Sociol. Methods Res..

[28] Schwarz G. (1978). Estimating the Dimension of a Model. Ann. Stat..

[29] Alangari A.A. (2014). Corticosteroids in the treatment of acute asthma. Ann. Thorac. Med..

